# In Vivo Correlation Between Macular Pigment Optical Volume and Retinal Layers Thickness

**DOI:** 10.1167/iovs.65.8.23

**Published:** 2024-07-15

**Authors:** Mariano Cozzi, Marco Casaluci, Giada Ruggi, Matteo Airaldi, Francesco Romano, Alice Bertoni, Marina Green-Gomez, John M. Nolan, Giovanni Staurenghi, Alessandro Invernizzi

**Affiliations:** 1 Eye Clinic, Department of Biomedical and Clinical Sciences, Luigi Sacco Hospital, University of Milan, Milan, Italy; 2 Nutrition Research Centre Ireland, School of Health Science, Carriganore House, Waterford Institute of Technology, West Campus, Waterford, Ireland; 3 The University of Sydney, Save Sight Institute, Discipline of Ophthalmology, Sydney Medical School, Sydney, NSW, Australia

**Keywords:** macular pigment, macular pigment optical volume, optical coherence tomography, fundus autofluorescence, outer nuclear layer, retinal layers thickness, early treatment diabetic retinopathy study grid

## Abstract

**Purpose:**

This study aims to investigate the potential in vivo relationship between macular pigment (MP) and retinal layers thickness in healthy subjects and dry, non-advanced age-related macular degeneration (AMD).

**Methods:**

An observational, cross-sectional study was conducted. Healthy subjects >40 years and patients with early or intermediate AMD were recruited. Structural OCT and macular pigment optical volume (MPOV) were collected for each subject. Retinal layers parameters were calculated based on the standard early treatment diabetic retinopathy study (ETDRS) map. Additionally, MPOV within 1°, 2°, and 9° of eccentricity was assessed and associated with retinal layers thickness and volume. Linear mixed-effects models were used to test the relationship between MP and structural OCT parameters, while adjusting for known possible confounding factors.

**Results:**

A total of 144 eyes of 91 subjects (60.4% females) were evaluated, comprising 43% normal eyes and 57% with early/intermediate AMD. Among the retinal layers, only the outer nuclear layer (ONL) thickness and volume appeared to be associated to higher MP levels. Specifically, the central ONL thickness was identified as a significant predictor of the MPOV 1°(*P* = 0.04), while the parafoveal ONL thickness (inner ETDRS subfield) was identified as a significant fixed effect on the MPOV 9° (*P* = 0.037). Age and the presence of drusen or subretinal drusenoid deposits were also tested without showing significant correlations.

**Conclusions:**

Among the retinal layers examined, only the ONL thickness demonstrated a significant association with MPOV. Consequently, ONL thickness might serve as a potential biomarker related to MP levels.

Lutein, zeaxanthin, and meso- zeaxanthin are xanthophylls exclusively concentrated at the macula, where they are referred to as *macular pigment* (MP).^1^^–^^3^ These molecules are primarily localized in the membrane of photoreceptor layers and at the level of retinal membranes surrounding the Henle's fibers.^4^ A physiological MP distribution peaks from the foveal central bouquet radially oputward in the inner plexiform layer (IPL), and nerve fiber layer.^5^^–^^7^ The role of MP has been extensively investigated over the last decades and extends from studies into the pigments optical properties to the association with antioxidant and anti-inflammatory benefits.^8^^–^^10^

There is an established consensus that individuals with higher MP levels exhibit better visual function,^11^^,^^12^ and that enrichment of MP results with improvements in visual function. Several studies speculated about the potential protective role of carotenoids that comprise MP against age-related diseases,^13^^,^^14^ in particular age-related macular degeneration (AMD).^15^ For all these reasons, a reliable way to measure in vivo MP levels in the human retina is critical.

Several methods are currently used to measure the MP.^16^^–^^18^ A recent standardized procedure for the assessment and report of MP has been proposed by Green-Gomez and coworkers^19^ using the dual-wavelength autofluorescence technique. This approach produces an accurate, valid, and reliable representation of the MP within the macula, which the authors report as MP optical volume (MPOV). Despite the great performance of this methodology, the dual-wavelength autofluorescence remains a research tool not yet applicable in routine clinical practice. The primary constraint lies in its exploratory nature, consequently resulting in less intuitive, standardized, and cohesive data outcomes.

Optical coherence tomography (OCT) is an established non-invasive technology that enables eyecare specialists to obtain retinal sections from the macular region with an axial resolution typically ranging from 5 to 7 µm. Recent software advancements and more accurate segmentation algorithms have contributed to obtain a precise retinal layer's segmentation with the opportunity to create thickness maps for each retinal layer.^20^

Various studies have investigated the role of MP by analyzing the cross-sectional images from OCT in living tissue focusing on different aspects.^21^^–^^23^ However, the relationship between the amount of MP obtained using the current standardized MPOV procedure and the thickness of certain retinal layers requires further exploration. In particular, an integrative approach combining recent technologies applied to a wider study population may help address a critical gap in the clinically applicability of measuring MP.

The purpose of this study is to investigate the relationship between MPOV using the dual-wavelength autofluorescence technique and the thickness distribution of various retinal layers assessed by spectral domain OCT (SD-OCT) in both healthy eyes and eyes affected by early and intermediate AMD. Establishing a potential anatomical correlation in vivo would enhance our understanding of MP bioavailability and pathophysiology and likely permit us to use retinal layers thickness as an indirect biomarker of MPOV.

## Methods

### Participants

This observational, cross-sectional study enrolled patients with early or intermediate AMD and healthy subjects who presented to the Eye Clinic of Luigi Sacco Hospital, University of Milan, from May 2022 to February 2023. The study adhered to the tenets of the Declaration of Helsinki and was approved by the local Ethics Committee. All participants were required to sign a written informed consent before enrollment.

Clinical examination and multimodal imaging were used to screen for potential subjects. Patients were included if they were at least 55 years old with evidence of drusen and or subretinal drusenoid deposits (SDDs) in one or both eyes that were detected during routine examination. Moreover, healthy subjects over 40-year-old with no evidence of macular diseases were included. For inclusion, visual acuity had to be ≥80 ETDRS (Early Treatment Diabetic Retinopathy Study) letters score (Snellen equivalent of 25/20 or better). If both eyes met the inclusion criteria, the two of them were included in the study.

Exclusion criteria were as follows: any evidence of advanced AMD (i.e., macular neovascularization or geographic atrophy) in either eye, a refractive error >±3.0 D or axial length >26 mm, the presence of other retinal or choroidal disorders, significant media opacities that compromised the quality of the fundus images, cataract surgery within the last six months, and any history of posterior segment surgery. Moreover, subjects who took or were taking oral carotenoid supplements were not included in the study. Two internationally recognized medical retina and imaging specialists (AI and GS) reviewed the images and patient records to confirm that participants met the eligibility criteria.

### Study Groups

Eyes were firstly divided into two different groups based on the clinical examination and imaging findings: healthy eyes and early/intermediate AMD eyes. The latter was further divided into drusen eyes (with presence of any drusen >63 µm) and eyes with SDDs, which may also exhibit drusen. Subretinal drusenoid deposits were identified as at least five definitive lesions on OCT and confirmed by the en face imaging modality.^24^ The Beckmann classification was employed to define AMD, whereas the inclusion of SDD was based on their recognition as a distinct AMD phenotype.^24^^–^^28^ Lens status was also recorded as phakic or pseudophakic based on the presence of the artificial intraocular lens (IOL) implants.

### OCT and MPOV Measurement

All subjects underwent a complete ophthalmic examination, including measurement of best-corrected visual acuity, slit-lamp biomicroscopy, fundus examination, and multimodal imaging. Before the imaging section, pupil dilation was obtained with 1% tropicamide and 2.5% phenylephrine. Imaging was performed with a fixed protocol in all patients. Both the SD-OCT scans and the dual-wavelength autofluorescence images were collected using a customized confocal scanning laser ophthalmoscopy Heidelberg Spectralis OCT (Heidelberg Engineering GmbH, Heidelberg, Germany). The SD-OCT acquisition protocol consisted of a 20° × 20° volume scan containing 49 horizontal B-scans with a resolution of 1024 × 48 pixels (each of those with a resolution of 1024 A-scan) and an averaging of 16 scans per B-scan to reduce speckle noise.

The dual-wavelength autofluorescence was used to obtain the MPOV at difference radii. The extended protocol has been described in details elsewhere.^19^^,^^29^ In brief, a 30-second movie of simultaneous blue (488 nm) and green (518 nm) autofluorescence of the central field was recorded for each eye. The MP density map was then generated by combining the results of the two wavelengths.

### Imaging Analysis

**Figure 1.**
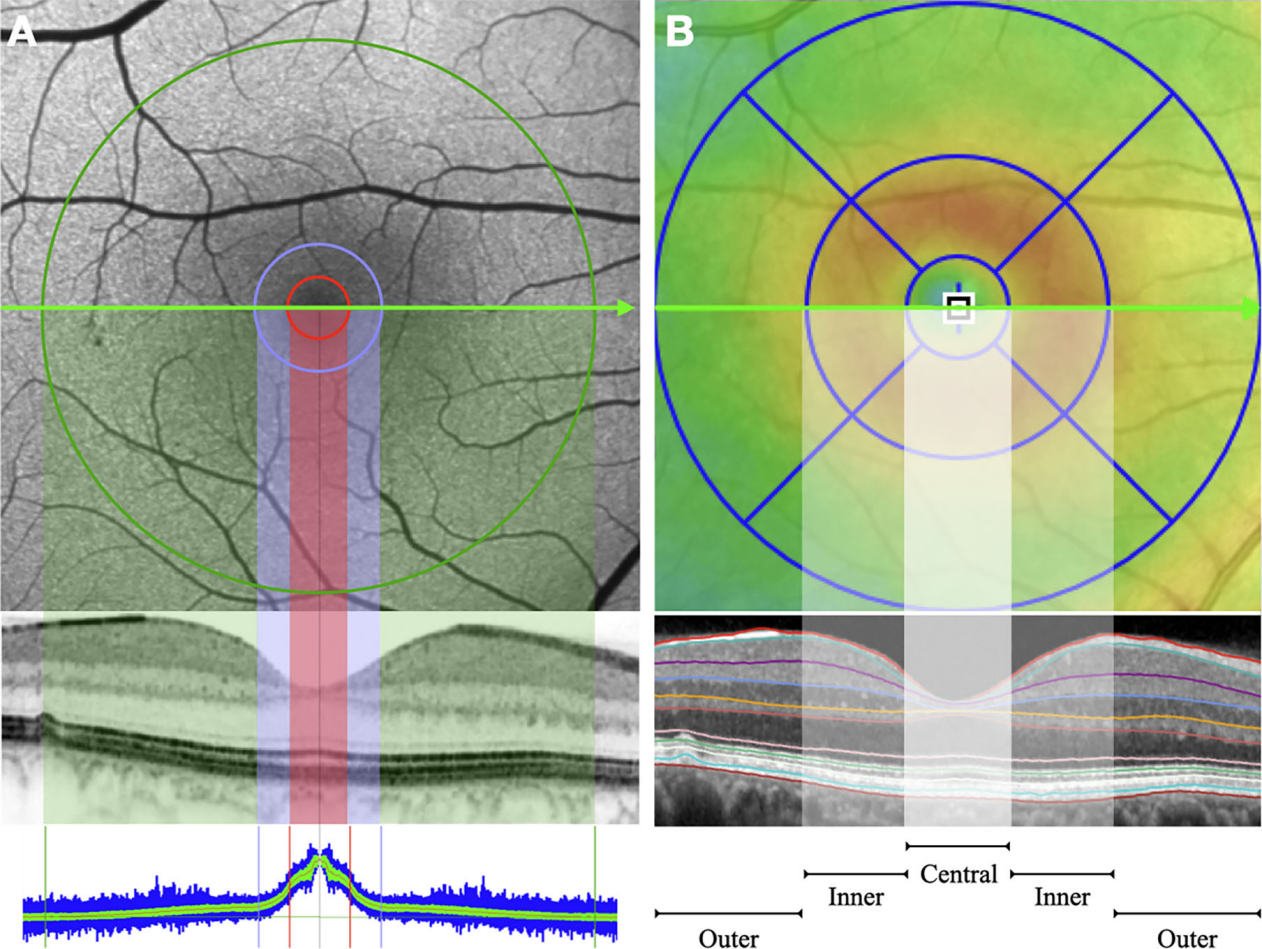
Macular pigment optical volume and thickness retinal layers. This graphic representation provides detailed correlation between MPOV and OCT. (**A**) Fundus autofluorescence image with three concentric rings superimposed at eccentricity of 1°, 2° and 9°. The area within the *red circle* represents the MPOV 1°, the area within the *blue circle* represents the MPOV 2°, and the area within 9° represents the MPOV 9°. The corresponding OCT B-scan illustrate the structural projection of the three areas of MP considered in the study. The bottom colored-code graph shows the spatial profile of the macular pigment in the same patient. (**B**) The same macular 6 × 6 millimeters area is now presented with the standard ETDRS grid superimposed. The three concentric rings delimitate the central subfield (1 mm diameter) surrounding by an inner and outer ring. The corresponding OCT b-scan illustrates the difference segmentation boundaries automatically applied by the software.

Heidelberg Engineering HEYEX version 6.3.2 was used to automatically calculate the overall retinal thickness in the central and inner ETDRS subfields, as well as the thickness of the nine retinal layers using the fully automatic embedded algorithm (Fig. 1). Each retinal layer segmentation was manually checked to correct any segmentation artifact if present. The overall ETDRS volume for each retinal layer was also obtained by the built-in software and considered for in the analysis.

The Spectralis investigational macular pigment optical density (MPOD) module was used to compute the averaged volume and obtain the MPOV. In short, the MPOV represents the numeric integration of all MPOD values within a given area delimitated by the circumference of a chosen eccentricity. To do that, a reference eccentricity has to be defined by the user. For the purpose of this study, the MP values were normalized by setting the MPOD values at eccentricity 9° to zero. This approach is consistent with the latest MPOV publications.^30^^,^^31^ The MPOV within a given area of 1°, 2° and 9° eccentricities were analyzed and correlated with the retinal layers thickness and volume obtained by SD-OCT (Fig. 1).

### Statistical Analysis

Descriptive statistics were presented as mean ± standard deviation (range) or frequency (%) as applicable. The distribution of quantitative variables was assessed using graphic Q-Q plot inspection.

The Sample size was calculated using pwr package for both correlation and general linear model. Considering a significance level (α) of 0.05 and a power of the test (1 − β) set as 0.8, we estimated a sample size of 122 eyes to determine a medium correlation level (*r* = 0.3) and a medium effect size with 5 predictor variables comprised in the linear mixed-effects model.

Pairwise comparisons were conducting using Bonferroni correction following multiple regression analysis to test differences in MPOV between groups, with age included as a covariate. The Kenward-Roger method was used to estimate the degrees of freedom in the analysis, ensuring accurate adjustment for the complex structure of the mixed-effects model.

The Pearson correlation coefficient (*r*) was tested to measure the correlation between the MPOV variable at 1°, 2°, and 9° of eccentricity (MPOV 1°, MPOV 2°, and MPOV 9°) with retinal layers thickness evaluated at the central one mm ETDRS subfield and at the inner ETDRS ring. Moreover, the total ETDRS volume from multiple retinal layers was also correlated with MPOV 9°.

Considering the outcomes of the retinal layers thickness and MPOV correlation analysis and the variables known to potentially affect MPOV biologically, linear mixed-effects models were used to estimate the expected MPOV. In particular, a first model used as main predictors the central outer nuclear layer (ONL), age and disease group as fixed effects and random intercept of eyes and participants as random effect. A second model was designed to estimate the expected MPOV 9° with ONL inner ring thickness, central ONL thickness, ONL volume, age and disease group as fixed effects and random intercept of eyes and participants as random effect. This allowed to control for potential correlations within the subject due to the hierarchical structure of the data.

All statistical analyses were performed using R software version 4.1.1 (a language and environment for statistical computing. R Foundation for Statistical Computing, Vienna, Austria. URL https://www.R-project.org/). Statistical significance was set at *P* < .05.

## Results

The study cohort was composed by 175 eyes from 112 subjects who met the inclusion criteria. Thirty-one eyes from 21 patients were excluded from the final sample due to poor quality images. The final analysis was hence performed on 144 eyes from 91 patients (60.4% females). Table 1 presents a summary of demographics and ocular characteristics of the study sample.

**Table 1.** Baseline Demographic and Ocular Characteristics

Characteristics	Overall (*n* = 144)	Healthy (*n* = 62)	Drusen (*n* = 55)	SDD (*n* = 27)
Age (yr)				
Mean ± SD	67.37 ± 11.1	61.08 ± 10.9	69.94 ± 8.6	76.5 ± 7.1
Median [minimum, maximum]	69 [42, 88]	61.5 [44, 80]	71 [55, 83]	76 [66, 88]
Gender				
Female	87 (60.4%)	34 (54.9%)	33 (60%)	20 (74.1%)
BCVA, ETDRS score				
Mean ± SD	84 ± 2.3	84.9 ± 1.6	83.8 ± 2.7	82.3 ± 2
Median [minimum, maximum]	84 [80, 91]	85 [81, 88]	85 [80, 91]	82 [80, 88]
Refraction, spherical equivalent				
Mean ± SD	−0.1 ± 1.3	−0.3 ± 0.9	0.2 ± 1.6	−0.2 ± 1.2
Median [minimum, maximum]	0 [−3.00, +2.75]	0 [−2.75, +1.50]	0.5 [−3, +2.75]	−0.5 [−2.75, +2.50]
Lens status				
Phakic	120 (83.3%)	58 (93.5%)	47 (85.5%)	15 (55.6%)

BCVA, best-corrected visual acuity.

Eyes were divided into the two groups. Sixty-two were classified as healthy, with a mean ± SD age of the subjects included in this group being 61 ± 10.9 (range 44–80) years. The remaining 82 had early/intermediate AMD, and the mean age ± SD in thin group was 72.4 ± 7.9 (range 52–88)

Healthy eyes were significantly younger than those with early/intermediate AMD (*P* < .001). The early/intermediate AMD group was further split in two subgroups depending on the presence of drusen and SDDs. Among the SDD group, seven eyes (26%) exhibited SDD only, while the remaining 20 eyes (74%) demonstrated SDD in conjunction with drusen. Detailed demographic data are provided in Table 1.

### MPOV Variation Between Groups

The mean ± SD MPOV 1° and MPOV 2° of the entire population were 1332.32 ±365.7 units and 3716.18 ±1209.1 units, respectively, whereas the MPOV 9° including an area of 9° radius was 14729.03 ±5457.3 units. The pairwise comparisons analysis of MPOV levels revealed no statistically significant differences between the three groups (Table 2).

**Table 2.** Multiple Regression Analysis With Pairwise Comparison

	Difference	Lower 95%	Upper 95%	Corrected *P*
MPOV 1				
Drusen-Healthy	87.0	−117	291	0.765
Drusen-SDD	−46.3	−299	206	1.000
Healthy-SDD	−133.3	−423	156	0.658
MPOV 2				
Drusen-Healthy	53.7	−567	675	1.000
Drusen-SDD	37.6	−747	822	1.000
Healthy-SDD	−16.1	−925	893	1.000
MPOV 9				
Drusen-Healthy	567	−2202	3336	1.000
Drusen-SDD	2676	−865	6217	0.135
Healthy-SDD	2109	−2018	6237	0.521

Results are averaged over the level of age. Degrees-of-freedom method: kenward-roger. *P* value adjustment: Bonferroni method for three tests.

### Correlation Between MPOV and Retinal Layers Thickness Within 1° and 2° of Eccentricity

**Figure 2.**
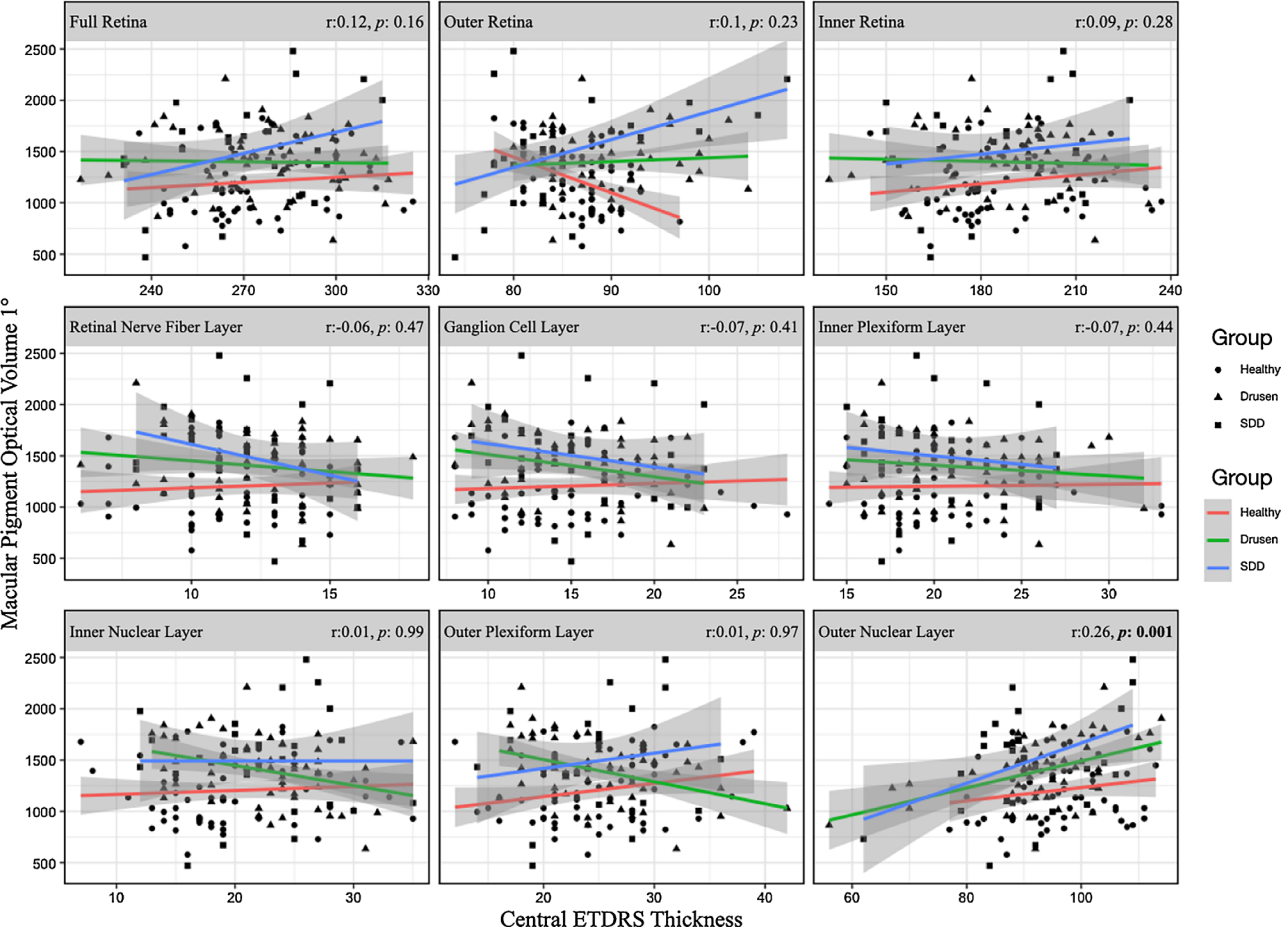
The relationship between MPOV 1° and the nine retinal layers thickness automatically quantified at the central ETDRS, tested across the entire study population. The Pearson correlation coefficient (*r*) is reported with the corresponding p-value for each retinal layer thickness. The figure also represents data distribution divided by disease group (healthy and early/intermediate age-related macular degeneration). It is worth noting that a significant linear positive relationship is presented between the MPOV 1° and the outer nuclear layer thickness (*r* = 0.26, *P* = 0.0018).

When considering MPOV values and retinal layers thickness in the whole population, the ONL was the only layer that exhibited a significant correlation with the macula pigment. The MPOV 1° and the ONL thickness obtained from the central ETDRS millimeter ring showed a moderate positive correlation, *r*(144) = 0.26, *P* = 0.002 (Fig. 2). A similar trend was observed between the MPOV calculated within 2 degrees of eccentricity and the central ONL thickness, with correlation of *r*(144) = 0.25, *P* = 0.002. None of the other segmentations showed significant correlations with the MPOV. In the first linear mixed model assessing the effect of age, disease group, and ONL thickness on the MPOV 1°, we found that the ONL central thickness was the only variable with a significant effect (*β* = 5.30, SE = 2.56, *P* = 0.04) (Table 3).

**Table 3.** Fixed Effects From the Linear Mixed-Effects Model Applied to MPOV 1°

Fixed Effects	Estimate	Standard Error	Pr(>|t|)
Intercept	378.51	346	0.278
Central ONL thickness	5.30	2.56	**0.040**
Drusen	108.30	74.31	0.147
SDD	162.58	104.90	0.124
Age	5.62	3.66	‘0.129

### Total MPOV

The MPOV calculated within 9° of eccentricity exhibited a significant correlation with the ONL central ETDRS millimeter thickness, *r*(144) = 0.26, *P* = 0.002. The MPOV 9° was also compared to the total volume of each retinal layer. We found a significant positive correlation between the MPOV 9° and the ONL volume within the entire ETDRS grid, *r*(144) = 0.29, *P* < 0.001.

To further explore the relationship between the MPOV 9° and the thickness of each retinal layer beyond the central ETDRS millimeter, we examined the average thickness of the four subfields of the inner ETDRS ring in relation with the MPOV 9°. Among these comparisons, the only significant correlation observed was between the MPOV 9° and the ONL thickness of the inner ETDRS ring, *r*(144) = 0.34, *P* < 0.001.

In our second linear mixed-effects model, we investigated the influence of different variables on the MPOV 9° Our findings revealed that the inner ONL thickness had a significant effect (*β* = 60.31, SE = 28.34, *P* = 0.037) on the MPOV 9° whereas neither the central ONL thickness nor the total ONL volume showed a significant impact on the MPOV 9°. These associations were not affected by age and disease group (Table 4).

**Table 4.** Fixed Effects From the Linear Mixed-Effects Model Applied to MPOV 9°

Fixed Effects	Estimate	Standard Error	Pr(>|t|)
Intercept	3736.66	6155	0.545
Parafoveal ONL thickness	60.31	28.44	**0.037**
Central ONL thickness	10.06	34.41	0.788
ONL Volume	−5596.53	4882.53	0.254
Drusen	917.27	1030.70	0.375
SDD	−2263.46	1516.74	0.138
Age	112.18	56.61	‘0.051

## Discussion

In this study we investigated the relationship between single retinal layers thickness and MPOV at different degrees of eccentricity. We demonstrated that MPOV was directly correlated with the ONL thickness while no association was found with other retinal layers thickness. Notably, the central ONL exhibited a correlation with the MPOV 1° whereas the overall MPOV out to 9° eccentricity was correlated with the thickness of the ONL at the level of the inner ETDRS ring.

The pairwise comparison, conducted through multiple regression analysis and accounting for age, demonstrated that MPOV was not significantly influenced by the presence of drusen or SDD. Conversely, Kar and coauthors recently reported higher level of MPOV in early and intermediate AMD compared to healthy controls.^7^ Of interest, our study was not specifically designed for investigating this aspect, and as such, the results should be interpreted accordingly. More recently, the same research group further illustrated similar findings based on the baseline data from the Alabama Study on Early Age-related Macular Degeneration 2.^30^ In a cohort of 809 eyes, they observed higher levels of MPOV within 2 and 9 degrees of eccentricity in AMD patients. These findings are of particular interest because previous studies conducted with different technologies demonstrated an opposite tendency.^32^^,^^33^ It remains challenging to clarify the reason why the MP, which is expected to play a protective role against oxidative stress, would be higher in eyes with an initial stage of the disease. One hypothesis suggested that xanthophyll transfer associated with soft drusen biogenesis may serve as possible triggering mechanism involving the localization of MP.^30^ Based on this hypothesis, xanthophyll transfer could contribute to drusen formation. However, further evidences are needed to elucidate this theory.

Xanthophyll carotenoids are known to be abundant in the central ETDRS millimeter subfield and tend to reduce toward the inner ring.^6^^,^^7^ Consequently, we first investigated the association between foveal MP and central ETDRS retinal layers. We were able to demonstrate, a significant association between MP and the ONL thickness. Our findings indicate that higher levels of MPOV are influenced by the ONL thickness. Notably, it is worth considering the possibility of an inverse relationship, wherein ONL thickness may also be influenced by macular pigment—a perspective that warrants further investigations. These results are consistent with previous speculations made by other research groups based on histologic studies.^5^^,^^34^

**Figure 3.**
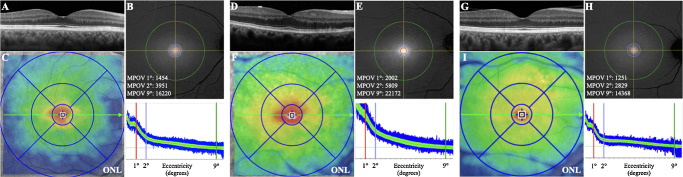
Example of three subjects included in study. The first case represents a healthy subject (**A**–**C**), the second case shows a patient affected by subretinal drusenoid deposits, (**D**–**F**) and the third case presents a patient with macular drusen only (**G**–**I**). The figure demonstrates a thicker ONL in the second patient (**D**, **F**) compared to the first (**A**, **C**) and third case (**D**, **F**). The color-coded thickness map of the ONL (**C**, **F**, **I**) further confirms the difference between the three subjects. Interestingly, the en face visualization of the MPOV, normalized by the ratio at 9° eccentricity, highlights higher levels of MPOV in panel **E** compared to panel **B** and **H**. This finding is further supported by the values of MPOV at 1°, 2°, and 9° of eccentricity, as well as by the presentation of the eccentricity graph at the bottom of panels **B**, **E**, and **H**.

Over the last two decades, advancements in technology have allowed for a detailed study of the spatial profile of MP and foveal metrics through improved MP measurement techniques and OCT scans with progressively higher resolution.^21^^,^^31^^,^^35^ Studies have shown correlations between foveal width, architecture, thickness, and the MPOD at certain degrees of eccentricity, even though with conflicting results. With the introduction of OCT Angiography, the foveal avascular zone has been linked to the MP spatial profile and central foveal thickness.^36^ Moreover, MPOD distribution have been significantly associated with retinal thickness and fovea pit profile.^37^ Absolute values of MPOD, as assessed with a macular pigment densitometer screener, have been found to correlate with central foveal thickness and neuroretinal volume. Furthermore, correlations between MPOD and the volume of certain retinal layers have been described: ganglion cell layer, IPL, and ONL.^23^ Our data further support the hypothesis that the central bouquet of cones, which mainly corresponds to the foveal ONL on OCT, is the primary element influencing the density of the central MP, even more when calculated as MPOV (Fig. 3).

Although a simple correlation was found between the overall MPOV and the ONL at different locations, we found in the multivariate analysis that the ONL thickness in the inner ETDRS map ring (corresponding to the parafoveal area) remains significantly correlated to the MPOV within 9° of eccentricity after correcting for confounding factors. Similar findings were reported by Nagai et al.^23^ who demonstrated a positive correlation between the full retinal volume at the level of the inner ETDRS ring and MPOD. It is important to highlight that MPOD reflects a distinct approach to quantify the amount of MP in living tissue, considering only the pigment's density across a specific degree of eccentricity without accounting for the volume within the boundaries. For this reason, our results require careful interpretation, as we compared, for the first time, a relatively novel approach to obtain MP with single retinal layers thickness assessed by SD-OCT.

A plausible reason to explain our findings may be attributed to the Henle fiber layer (HFL). First, HFL contains the highest level of MP in the human body.^5^^,^^34^ Second, because of its unique orientation and packing geometry, HFL exhibits an optical property known as birefringence, a refractive index that basically depends on light incidence direction.^38^ Consequently, HFL are not visible in a standard OCT b-scan, but they appear, partially within the ONL, when a directional OCT acquisition is employed.^39^^,^^40^ The parafoveal ONL thickness, measured with standard OCT, necessarily includes both the ONL and HFL, and thereby corelates with the MP bioavailability.

When we examined the correlation between the MP and the different retinal layers thickness, we found a significant relationship only with the ONL. This finding was observed across all three different MPOV sectors included in the analysis. By contrast, previous studies have reported a significant correlation between the overall retinal thickness and MP.^22^^,^^23^ Additionally, positive correlations between MPOD and ganglion cell layer and IPL volume have been described in a Japanese population.^23^ We believe that relationships involving OCT thickness of retinal layers of approximately 20 µm might be challenging to establish with the current technology. Future studies with higher OCT resolution are warranted to gain a deeper understanding of other retinal layers’ thickness involved in MP concentration.

There are some limitations to this study that we want to acknowledge. First, we pooled our cases into a single unbalanced cohort for statistical purposes. The primary aim of the study was to investigate the relationship between MP and OCT retinal layers parameters, regardless the underlying macular condition. This approach was also adopted in previous studies.^7^^,^^41^^,^^42^ Second, we intentionally excluded patients who were under carotenoids oral supplementations to reduce any possible external influence on MP levels. Although this approach resulted in a more homogeneous study group, it would be interesting in future research to investigate the impact of oral supplementation on the relationship between MP and retina layers thickness. Conversely, the primary strength of the study lies in the relative novel approach used to quantify the MP. Although most studies in the last three decades aimed to describe the spatial profile and distribution of the MP, we calculated the MP volume within a given area delimitated by the circumference of a chosen eccentricity, in accordance with the most recent guidelines.^19^

In conclusion, the findings of this study suggest a significant association between the ONL thickness and the quantity of macular pigment measured in vivo using the dual-wavelength fundus autofluorescence approach. The HFL, recognized as the retinal structure richest in macular pigments, is normally not detectable on standard OCT scans and is likely the main responsible to the identified correlation. These results contribute valuable insight into the relationship between MP and retinal anatomy, highlighting the importance of ONL thickness as potential biomarker for macular pigments levels.
